# Consumers’ Preferences for Electronic Nicotine Delivery System Product Features: A Structured Content Analysis

**DOI:** 10.3390/ijerph14060613

**Published:** 2017-06-07

**Authors:** Christine E. Kistler, Trisha M. Crutchfield, Erin L. Sutfin, Leah M. Ranney, Micah L. Berman, Gary A. Zarkin, Adam O. Goldstein

**Affiliations:** 1 Department of Family Medicine, School of Medicine, University of North Carolina at Chapel Hill, 590 Manning Drive, Chapel Hill, NC 27599, USA; leah_ranney@unc.edu (L.M.R.); adam_goldstein@med.unc.edu (A.O.G.); 2 Lineberger Comprehensive Cancer Center, University of North Carolina at Chapel Hill, Chapel Hill, NC 27599, USA; trisha@unc.edu; 3 UNC Center for Health Promotion and Disease Prevention, University of North Carolina at Chapel Hill, Chapel Hill, NC 27599, USA; 4 Department of Social Science and Health Policy, Wake Forest University, Winston-Salem, NC 27599, USA; esutfin@wakehealth.edu; 5 College of Public Health & Moritz College of Law, Ohio State University, Columbus, OH 43210, USA; mberman@cph.osu.edu; 6 Behavioral Health and Criminal Justice Research Division, RTI International, Research Triangle Park, NC 27599, USA; gaz@rti.org

**Keywords:** electronic nicotine delivery system, qualitative, consumer preferences

## Abstract

To inform potential governmental regulations, we aimed to develop a list of electronic nicotine delivery system (ENDS) product features important to U.S. consumers by age and gender. We employed qualitative data methods. Participants were eligible if they had used an ENDS at least once. Groups were selected by age and gender (young adult group aged 18–25, *n* = 11; middle-age group aged 26–64, *n* = 9; and women’s group aged 26–64, *n* = 9). We conducted five individual older adult interviews (aged 68–80). Participants discussed important ENDS features. We conducted a structured content analysis of the group and interview responses. Of 34 participants, 68% were white and 56% were female. Participants mentioned 12 important ENDS features, including: (1) user experience; (2) social acceptability; (3) cost; (4) health risks/benefits; (5) ease of use; (6) flavors; (7) smoking cessation aid; (8) nicotine content; (9) modifiability; (10) ENDS regulation; (11) bridge between tobacco cigarettes; (12) collectability. The most frequently mentioned ENDS feature was modifiability for young adults, user experience for middle-age and older adults, and flavor for the women’s group. This study identified multiple features important to ENDS consumers. Groups differed in how they viewed various features by age and gender. These results can inform ongoing regulatory efforts.

## 1. Introduction

Electronic Nicotine Delivery Systems (ENDS) emerged on the United States market in 2007 [1]. Since then, ENDS use has risen substantially [2]. As of 2014, a third of current smokers reported having used ENDS [3,4]. In response to public health concerns, the FDA issued a regulation deeming ENDS to fall under its authority and imposed some restrictions on ENDS manufacturing, sales, and marketing [5]. Moreover, ENDS-related laws have already been enacted in 34 states [6], and additional regulation is anticipated. Knowing how potential ENDS features, such as perceived health effects and cost, impact decisions to use ENDS devices across age groups and gender, may inform the development of future regulations [4].

There is ongoing debate about the potential public health effects of ENDS. Some individuals believe ENDS may be effective at reducing the harms caused by tobacco cigarettes for those unable to quit completely, while others believe it may serve as a “gateway” to tobacco cigarettes [7,8]. Ultimately, the public health impact of ENDS will depend on who uses the products, how they use them, and for what purpose [4]. For example, even if ENDS prove to be much less harmful than cigarettes, recreational use of ENDS by younger consumers may still be problematic if it promotes smoking initiation, prolongs ongoing smoking, or leads to dual and poly use of other tobacco or drug products [7,9,10]. Regulations targeting ENDS features that appeal to young adults could prove effective, particularly if the targeted features are not as salient for older adults who may seek to transition away from tobacco cigarettes [11,12,13]. Information about consumers’ preferences for ENDS features will be important however the final evidence on the public health effects settles out.

ENDS have many different features that may impact consumer purchase decisions. Research shows user experience [14,15,16], social acceptability [17], and perceived health benefits [12,18,19,20] influence ENDS use in adults. A study of 915 Canadians found that the potential use of ENDS for tobacco cessation was one of the most important features affecting intended use [21]. Additional research has found the preferences placed on ENDS features including flavors [21,22] and nicotine content [21] vary by gender and age. Similar to differences found among users of traditional tobacco, a recent study of ENDS users found significant differences in user experience, flavors used, and nicotine content, in how men and women use ENDS [23]. Another study found men perceived tobacco-flavored ENDS to be less harmful than did women [21]. Additionally, ENDS companies appear to be using some of the same media strategies such as an advertising focus on independence, sexuality, and sociability to market ENDS devices to women just as they have used to promote traditional tobacco products to women [24].

Other differences have been seen between young adults and older adults. One study found that availability of a significant number of flavors was more likely to increase ENDS use in younger adults than older adults [25]. Studies of adult ENDS users have found a variety of reasons for use, and have shown that younger adults report using ENDS for different reasons than older adults [26,27,28]. Younger adults appear less likely to believe ENDS can be used for tobacco cessation or to actually use ENDS for smoking cessation as compared to older users [27,29]. However, we found no studies specific to older adult ENDS users’ preferences for ENDS features, though the literature suggests older smokers’ preferences differ from younger populations [30,31].

Prior studies only focused on a few ENDS features rather than a comprehensive list of features and how that list varies by age and gender. The evidence supporting the hypothesis that various consumer groups differ in how they prefer distinct ENDS features is incomplete. Though this evidence currently suggests a need to target specific groups to enhance the effect of ENDS regulations, further work is warranted. To better understand different consumer groups’ preferences for individual ENDS features, we conducted formative research through a series of focus groups and individual interviews with people who had tried ENDS at least once in their lifetime to create a comprehensive list of ENDS features.

## 2. Materials and Methods

### 2.1. Study Design

As part of a larger study to determine the importance of ENDS features on consumer use, we conducted focus groups and individual interviews using purposively sampled participants. The interview guide was developed based on the literature [16,17,21,26,32] and prior work by the University of North Carolina’s Communication for Health Application and Interventions (CHAI) Core. The CHAI core has extensive experience conducting focus groups in the tobacco use field [33,34]. A semi-structured focus group guide was used to encourage discussion, and probes were used to clarify and evoke additional detail (see Supplementary Materials). The guide was not pilot tested due to resource constraints. The first half of the group discussion, lasting about an hour, focused on generating discussion around perceptions of different ENDS products and perceptions on the importance of ENDS features (i.e., cost or flavors). A feature was broadly defined as any topic discussed by participants that they mentioned as important to their ENDS use. It could include tangible features of the device such as flavors; beliefs or perceptions about using the device such as social acceptability; or functions of the device such as smoking cessation. The second half of the focus group, lasting about 30 min, was spent having participants pre-test a draft survey of a discrete choice experiment designed to test the importance of ENDS features. The attributes used in the discrete choice experiment were developed from the literature and from the features identified as important in the prior focus groups. Participants also provided feedback on the survey’s usability and understandability. Three 90-min focus group discussions were conducted with 29 participants between April and June 2015, in a private conference room in a research center. The focus groups were recorded, and conducted by a three-person team—two moderators and a note taker. The two moderators (Russel Teal (RT) and Trisha Crutchfield (TC)) collectively have over 20 years of experience facilitating focus groups and interviews and analyzing qualitative data. One moderator is male, the other is female; one under 35, one over 35. Thus at least one moderator matched the focus group participants. No moderator matched the older interviewees, given their ages.

Given the paucity of older ENDS consumers, we could not recruit enough individuals to form a focus group; older adults were interviewed individually. We conducted five individual, 90-min, in-depth interviews with 68–80 year olds. The interviews were conducted and recorded either on the phone or in a comfortable location selected by the participant. The research team reviewed the focus group and interview notes after conducting each focus group and interview. Additionally, we could conduct only a limited number of focus groups, and we were able to enroll only one middle-age women’s group due to resource constraints. Thus, while it appeared that overall our domains had reached saturation, our results may not fully represent these populations. The research team believed data saturation had been reached because no new domains appeared. All participants consented and received $50 for participation. Consent and participation were voluntary. The Institutional Review Board at the University of North Carolina at Chapel Hill approved this research (IRB #14-2115).

### 2.2. Participants and Recruitment Procedures

Focus group and interview participants were recruited from central North Carolina through advertisements on social media (i.e., Facebook), distribution of flyers on local telephone poles, at local vape shops, retirement centers, restaurants, coffee shops, and through campus email listservs and word of mouth. Interested individuals completed an online screener or telephoned and were screened by CHAI core research staff to ensure their eligibility status. Individuals were invited to participate in a focus group or interview if they met the inclusion age criteria (young-adult aged 18–25; middle-aged 26–64; older adult aged 65+) and had tried an ENDS device at least once. We defined ages 26–64 as middle-aged given that this time period spans the working life of most adults and is discrete from the educational years of early adulthood on one end of the spectrum and retirement on the other. We chose to include older adults because up to 4% of the population has used ENDS products [35], and tobacco smoking in older age increases overall mortality [36]. Five percent of older adults quit tobacco cigarettes after age 60 and six percent continue to smoke into their 70s [36]. We chose a minimal threshold of ENDS use in order to elicit the most diverse group of consumers available. We scheduled approximately 15 eligible participants per focus group with the aim of having 7–10 participants be present the night of the focus group. Though a convenience sample of ENDS consumers in the local community, we purposively aimed to include as many minority participants as possible and an even distribution of men and women to allow a variety of diverse perspectives. Because there were a disproportionate number of men in the initial focus groups, a third group of middle-aged adult women was conducted. We collected basic demographic characteristics about the participants in the focus group and individual interviews.

### 2.3. Analysis

We used descriptive statistics to describe participant characteristics. We conducted a structured content analysis of the professionally transcribed audio files using ATLAS.ti (ATLAS.ti Scientific Software Development GmbH, Berlin, Germany) [37,38,39]. Audio files were not edited and personal identifiers were not used. To ensure consistency in coding, we developed a draft coding manual that defined each code conceptually, and described its application. The codebook was based on the focus group guide and from focus group notes. Two coders (RT and TC) independently coded a randomly selected focus group, marking the text with the applicable codes. Questions about the meaning of codes, the differences between codes, and the decision rules about when to apply codes were discussed and resolved through consensus during meetings over several weeks. The codebook evolved over time as new codes were added, and definitions were refined. The team discussed and reconciled the coding over several weeks. Then three coders (RT, TC, and Maihan Vu (MV)) independently coded the remaining focus group and interview transcripts. An incident for the analysis was any time a feature was mentioned, whether it be in a phrase, a sentence, or longer. For example, each time cost was mentioned within the conversation, it treated as an incident and was coded “cost”. If a feature was mentioned once, it would be coded once. The default size of a code was a paragraph. Inductive coding techniques as described by Strauss and Corbin [40] were used along with the constant comparison method [41]. Preferences for ENDS within and across participant groups were identified.

## 3. Results

Of the 34 participants in the focus groups and interviews, 67% were white, 59% female, and 44% had at least a four-year college degree, as shown in Table 1. Most were current smokers (65%) and current ENDS consumers (82%).

Participants mentioned 12 ENDS features important to their ENDS use, including: (1) user experience; (2) social acceptability; (3) cost; (4) health risks/benefits; (5) ease of use; (6) flavor; (7) smoking cessation aid; (8) nicotine content; (9) modifiability (10) ENDS regulation; (11) bridge between tobacco cigarettes; (12) collectability. The frequencies of the features mentioned and detailed definitions are shown in Table 2.

### 3.1. Most Common Features Overall

Overall, user experience, social acceptability, and cost were most frequently mentioned across ages and gender. While not as common, health risks/benefits were mentioned relatively often. ENDS regulation, nicotine content, use as a bridge between cigarettes, and collectability were mentioned less frequently among all groups.

When we examined the three most commonly reported features, all participants mentioned user experience as one of the most important features, encompassing both positive and negative experiences. A young adult said, “*I think people also like clouds, people chase clouds… that’s what attracted me*”. A middle-aged woman conversely said, “*I don’t think it’s as satisfying as a real cigarette… it’s like expecting a filet mignon and getting cube steak*”. In regard to social acceptability, a middle-aged participant said, “*I think it’s probably a little more socially acceptable to smoke an e-cigarette now… Everybody looks down on people who smoke*”. Participants also talked about the socializing that ENDS consumers can enjoy. One young adult said, “*You can go hang out in some of the stores, literally they are hubs…They got couches* [sic]*…You just walk in. You chill…It’s a nice community*”. Most participants felt that ENDS cost less than traditional cigarettes. An older adult said, “*I was spending $40 a week on cigarettes, so that’s $150 plus [a month]. And at the most I would say I’m spending…$50 a month… I was to the point where I was eating spaghetti, and noodles, and going to the food bank, and it was really hard*”. Participants who also mentioned collectability and modifiability tended to find ENDS costlier. A younger adult reported, “*Because I can go through 30 mg easily in two days on this because I’m blowing such big clouds that I’m going through juice constantly… This is a super expensive habit*”.

### 3.2. Most Common Features by Group

Differences in how frequently features were mentioned emerged between different age groups. Middle-age and older participants discussed user experience most often, with similar responses as noted above. However, younger adults mentioned modifiability most often, though some members noted it was “*more of a boy thing*” because men like to “*pull things apart*”. In general participants discussed changing out the batteries, particularly for the bigger devices, to modify electric current, replacing the coils and tank, as well as modifying the airflow through the mouthpiece. One younger adult said, “*Every e-cig has a coil like this on the inside of it no matter how big or small it is… you can take out that little piece that you can change this*”. While user experience was frequently discussed, the women’s group mentioned flavor most often. One woman said that, “*it almost becomes like a habit. Because you just think well, I’m getting up in the morning and I want strawberry this day. Tomorrow grape. Or vanilla, cherry*”. Other women mentioned being attracted to the names of the flavors, such as one woman who said, “*It was the same way with the unicorn milk; that name…And I said, ‘Ooh, let me try that’*”. Though not mentioned most often by any group, older adults mentioned using ENDS as a smoking cessation aid more than participants in the focus groups. An older adult said that, “*…you keep trying to quit but at your age you go back to it. This is not going to kill you, so if you want to vape, vape, and you can, you just don’t have to worry about quitting it*”. Another older adult reported that, “*I was smoking kind of heavy, went to my doctor, and he put it to me more or less, you can call the undertaker or you can quit smoking. Which one would you do? And I said, well, I really rather quit smoking, but it’s kind of hard to do. I’ve tried two or three times. He said, well, have you tried the e-cigarette? I said, no, sir*”.

## 4. Discussion

A structured content analysis revealed that consumers reported a diverse set of features as important; middle-age adults and older adults mentioned user experience most often, while young adults mentioned modifiability most often, and the women’s group mentioned flavor most often. Furthermore, our work is novel in that is suggests that interest in ENDS features likely differs by age and gender. Women favored flavor variety and older adults mentioned the smoking cessation aid potential of ENDS more often than other groups. Our results expand the knowledge base on ENDS preferences and use by generating a robust list of features that can be explored in future research and regulatory efforts.

These findings expand on prior ENDS research [12,14,15,16,17,18,19,21]. User experience, social acceptability, and cost were frequently mentioned across all groups. Regulations targeting these three features may have a significant impact on ENDS use, as the FDA has recently deemed ENDS within its regulatory authority. User experience was a relatively expansive feature and deserves further exploration to best determine which aspects of devices (such as their size, shape, or other functionalities) are most amenable to regulation. While many states have some forms of regulations, bans of ENDS indoors or in public places are rare, and additional regulations in these domains may disrupt its social acceptability [42]. As cost was an important feature of ENDS use, efforts to increase cost may disrupt use. Given the negative socio-economic gradient in tobacco smoking [43], cost effects on ENDS consumers with lower socio-economic status merit particular attention. Supporting this idea, is evidence from a recent study that found cost found significant price sensitivity for ENDS use in all adult age groups [25]. In sum, limitations on the type of devices sold, permissible locations to use, and taxation to increase the cost of ENDS products may all reduce ENDS use.

While research exists detailing different consumer groups’ ENDS use [44], few studies focus on different consumer groups’ preferences for different ENDS features. Our findings show that younger adults mention modifiability more frequently, which confirms prior work examining younger adult consumers’ preferences for ENDS [16,17]. Regulations regarding the manufacturing of ENDS that prevent or limit product modifiability could potentially be an effective way to reduce use among young adults without having as much of an impact on other groups, such as older adults [45]. Additionally, regulations that restrict the use of flavors (other than tobacco flavor) may decrease consumption especially in women. Previous studies among adult tobacco cigarette and ENDS consumers reported that a variety of flavors was important to the majority of consumers and influenced device choice [12,14,46]. It is increasingly likely that flavor plays an important role in decisions by women (and to a lesser degree by men) to use or continue using ENDS. More work is needed to understand how ENDS features impact ENDS use differentially by age and gender.

Although we conducted the research using standard research procedures, the results must be interpreted in light of several limitations. Foremost, our findings include a select sample of participants from a single geographic location and the results may not be applicable to other groups of ENDS users outside of our locale, or to adolescents or Asian-Americans. While our sample was relatively diverse in terms of racial distribution and education, we did not specifically examine differences by race or socio-economic status. Additionally, we were limited in the number of focus groups we could facilitate, and we were able to enroll one middle-age women’s group, but not a young adult women’s group, due to resource constraints. While we cannot say for certain if we reached thematic saturation within each of the groups, it appeared that overall our domains had reached saturation. However, our results may not fully represent the entire universe of features in these populations. Moreover, qualitative research can be subject to bias in subject selection, moderators, or biased questioning. Because of our focus on examining the breadth of features important to ENDS consumers, our results may not have accurately demonstrated the depth of participants’ feelings. Lastly, while we have found evidence for differences in ENDS features by age and gender, because we used a structured content analysis, our results are susceptible to issues of neutrality and trustworthiness inherent in the technique [39]. We did not control for other participant characteristics in our analysis, such as race, frequency of ENDS use, or dual-use status. We sought to reduce these biases through a thorough review of the literature prior to the development of our codes as well as frequent meetings to refine those codes, and the use of multiple coders to improve triangulation and grounding [47].

## 5. Conclusions

In conclusion, we found 12 ENDS features were prevalent in consumers’ discussions of ENDS use, but that these features differed by age and gender. Understanding how potential ENDS features impact ENDS use can facilitate the creation of effective policies and regulations at the national, state, or local levels.

## Figures and Tables

**Table 1:** Participants’ Tobacco Use Characteristics, *n* = 34.

Characteristics	Total, *n* = 34 (%)	Young Adult, *n* = 11	Middle-Aged Adult, *n* = 9	Women’s Group, *n* = 9	Older Adults, *n* = 5
Age, mean (SD)	41 (18)	23 (4)	48 (9)	38 (9)	73 (4)
Female	19 (56%)	2 (18%)	5 (56%)	9 (100%)	3 (60%)
Race					
White	23 (68%)	8 (73%)	4 (44%)	6 (67%)	5 (100%)
Black	9 (26%)	1 (9%)	5 (56%)	3 (33%)	0 (0%)
Other	2 (6%)	2 (18%)	0 (0%)	0 (0%)	0 (0%)
College degree or higher	15 (44%)	5 (45%)	5 (56%)	3 (33%)	2 (40%)
Mean age, first cigarette (SD)	17 (5)	15 (2)	18 (6)	14 (5)	20 (7)
Current tobacco cigarette user	22 (65%)	6 (55%)	7 (78%)	7 (78%)	2 (40%)
Smokes more than one pack/day	3 (9%)	0 (0%)	0 (0%)	3 (33%)	0 (0%)
First smoke within 30 min of waking	13 (38%)	4 (36%)	3 (33%)	5 (56%)	1 (20%)
Tobacco quit attempt in past 12 months	11 (32%)	2 (18%)	3 (33%)	3 (33%)	3 (60%)
Used ENDS in last 30 days	28 (82%)	9 (82%)	5 (56%)	9 (100%)	5 (100%)
Used ENDS daily in last 30 days	11 (32%)	5 (45%)	0 (0%)	3 (33%)	3 (60%)

**Table 2:** Frequency of ENDS features by group.

ENDS Features	Definition	Frequency of ENDS Feature				
		YA, *n* = 11	MA, *n* = 9	WMA, *n* = 9	OA, *n* = 5	Total
**User Experience**	The odor, feel, texture, appearance, taste, cloud blowing or any other experience directly related to using ENDS including their novelty.	11	37	17	53	118
**Social Acceptability**	The sense of encouragement or acceptability to use ENDS and to connect with others (camaraderie) who vape or conversely when individuals report increased stigmatization/decreased acceptability when using ENDS.	12	23	7	20	62
**Cost**	The amount an individual spends or pays for an ENDS or its components, including cartridges, replacements coils, batteries, or juice. This feature also included the ability to use coupons, comparison shopping, and bulk purchasing.	12	15	13	22	62
**Health Risks/Benefits**	Any health issues connected with using/not using ENDS in either a negative or positive way, including any mention of health risk, behavior changes or health effects of others, e.g., their children or other family members.	7	19	13	21	60
**Ease of Use**	The difficulty or ease to manipulate or use an ENDS poses to a consumer, including how difficult or easy it is to find/purchase/obtain ENDS or places where they can be used.	13	14	10	15	52
**Flavor**	The number and type of flavors purchased, the mixing of flavors, as well as the smell of flavors, including whether they are liked or disliked.	7	5	20	17	49
**Smoking Cessation Aid**	The ability of ENDS to assist in tobacco cigarette smoking cessation in a positive or negative way.	2	8	8	20	38
**Nicotine Content**	The amount of nicotine in an ENDS, including the ability to control the nicotine content in connection with the smoking cessation.	7	6	6	11	30
**Modifiability**	The capability to alter ENDS to meet their needs, including both favorable and unfavorable capabilities, excluding changing flavor “e-juice” cartridges.	14	3	6	1	24
**ENDS Regulation**	The federal, state, or local control of ENDS, including the desire for or fear of regulation including mention of existing laws.	3	4	4	2	13
**Bridge Between Cigarettes**	The use of ENDS as a ‘crutch’ or substitute when one is not able to smoke tobacco cigarettes to tide them over until the next tobacco cigarette.	4	3	2	0	9
**Collection**	The ability to purchase multiple ENDS and/or mention of shopping for different styles of ENDS, including flavors or other components.	3	0	2	0	5

## Supplementary Materials

The following are available online at www.mdpi.com/1660-4601/14/6/613/s1, S1: Moderator guide for focus groups for important characteristics of E-vapor product use among a Nationally Representative sample of adult smokers in the US (Kistler, PI).
