# Supplementary material for: Consumers’ Preferences for Electronic Nicotine Delivery System Product Features: A Structured Content Analysis
Source: Int J Environ Res Public Health. 2017 Jun 7;14(6):613. doi: 10.3390/ijerph14060613 (PMC5486299; doi:10.3390/ijerph14060613)
Supplement: Supplementary file 1 [file ijerph-14-00613-s001.pdf]

Moderator guide for focus groups for
Important Characteristics of E-vapor product Use among a Nationally Representative sample of adult smokers in the US (Kistler, PI)

Study Introduction *(5 minutes)*

Greeting
Welcome and thank you for being here today. We really appreciate you taking the time to participate in this research project. My name is ___________ and I will be the moderator for the first part of our discussion. This is Trisha, she will be the moderator for the second part of the discussion. Today we will be doing two things, first we will have a discussion about e-vapor products and then you will complete a survey about e-vapor product preferences. After you complete the survey, we will have a second discussion about what you think about the survey. We expect the two discussions will last about two hours total. We will give you a 5 minute break after the first part of the discussion. At that time, we will give you a chance to use the restroom. We ask that you not leave the building. At the end of the discussion we will provide you with a $50 check for your participation.

Role
Our role today will be to ask some specific questions and to keep the conversation going. We have a lot to cover, so we may need to change the subject or move ahead with the discussion. But, please stop us if you want to add anything or if you have any questions.

We are fortunate to have some help today. I'd like to introduce you to our co-moderator for the first part of the discussion, _____________. S/he may ask some clarifying questions as they come up.

The note taker for today is _____________. His/her job will be to take notes during the discussion. We want to be sure to get all of the important things you say.

Purpose
I think everybody knows why we're here, but let me go over it just in case. We are part of a research team at UNC. The main thing we are interested in today is hearing from you, the experts, about your ideas, opinions, and concerns as they relate to e-vapor product use and the reasons why you use them. Your participation in today's focus group will help us gain valuable insight into how individuals make decisions on whether to use these products or not.

I would like to state that our conversation is being audio-taped to help us remember what is said. You may ask me to turn off the recorder at any time or simply say you do not want to answer a question. Everything said here today will be confidential. Nothing you say will be connected with your name. We would also like everyone in this group to keep things confidential, so whatever you say and whatever you hear someone say, we hope that it will not go out of this room. I hope you will feel free to speak openly but also be aware of our limits in protecting your confidentiality.

How to Participate
Today you will be participating in a focus group. This will be a group discussion. It's not an interview where I ask a question and each person answers the question and we move on to the next one. Instead, we'll be putting topics on the table and the idea is for everyone to participate in the discussion with each other. It's particularly helpful if somebody says something and you're sitting here thinking, "Yeah, that's sort of the way I think about it or feel about it" and you share that. We also want to hear from you if you are thinking, "NO, that's not the way I do it. That's not how I think about it." We want to hear both similarities and differences within the group. There are no right or wrong answers to these questions.

Consent Process *(5 minutes)*

Pass out Consent Forms + pens

Before we begin the focus group, we need you to officially consent in writing to participate in this research study. Here are two consent forms. One you will sign in ink and give to us and the other you can take home for your records. Let's briefly go

over the consent document together. After we go over it together, you can read the entire document and ask us any questions if you like. **Review Consent Form**

START RECORDING
Part I (Total 70 minutes)

I. Establishing a context for using e-vapor products *(10 minutes)*

All of you were invited into this group because you have used or currently use e-vapor products. So let's start with some common ground. Let's go around the room and have everyone share the story of the first time you used an e-vapor product?

II. Perceptions on different e-vaping products devices *(7 minutes)*

By a show of hands, how many of you are carrying your device now? Can you show us?

What kind of electronic vaping device(s) do/did you use?

Do you use more than one device?

How did you decide between which devices to try? (like/dislike)

How long did it take you to find a device you like?

III. Perceptions on the importance of e-vapor product features *(44 minutes)*

What are some of the things you've heard others say about e-vapor products?

What are the reasons that you decided to try or use an e-vaping product?

[prompt on below features if they don't come up]

Cost
How has cost affected your use of e-vapor products?

What do you estimate you spend per week on e-vapor products (cartridges, drops)?

How much did the device cost that you use?

How did cost factor into the device(s) you use?

Did you buy a start-up package, where some part of the package was free or a reduced cost to get you to try the product?

User Experience
How do e-vapor products differ from a regular cigarette/other tobacco products in terms of your experience?

[prompt, if it doesn't come up] Odor? Feel (hits the throat like traditional cigarette aka Throat Grab; Makes your lungs feel a certain way)? Appearance? Taste? Buzz? Can make clouds? Do you value being able to blow vapor clouds?

Ease of use
Where and when do you usually use e-vapor products?

Tell me about how your use has been impacted, if at all, by the rules and regulations about where you can have an e-vapor products

What kind of push-back, if any, have you experienced from using an e-vapor products in public areas? At work? At home? At hotel rooms? Bars/Restaurants? Other?

Harm reduction
In what ways do e-vapor products affect your health, if at all?

What about the health of others?

[Prompt with these specific diseases/issues is they don't come up] Cancer? Heart disease/Heart attacks? Stroke? COPD? Lung diseases? Fire risk?

Smoking Cessation Aid
We have # people in this group. How many of you use only e-vapor products? Looks like # people. Now, how many also smoke tobacco products? Looks like # out of X people.

How have you used e-vapor products to reduce or stop smoking cigarettes/other tobacco products, if at all?

Flavor
What kind of flavors have you tried with e-vapor products?

Menthol?

Technical Capabilities
What technical features do you like most about your device(s)?

[Prompt with these specific features if they don't come up] Re-chargeable? Battery Powered/Duration of power? Does not require ashtray/matches/lighter? Multi-use?

Modifications
Do you value being able to modify your e-vaping device(s) to meet your needs?

[Prompt with these specific modifications if they don't come up] Modify power/voltage? Modify airflow/vapor? Adjust nicotine/flavor? Modify to use other substances?

Nicotine Content
How often do you use your e-vaping device?

On average how much nicotine do you use in your device if any?

Purchase Convenience
Where did you get your e-vaping device?

Where would you like to purchase e-vaping devices?

How would you feel about being able to get an e-vaping device with a prescription from a healthcare provider?
  What if your health insurance would cover it?

Side Effects & Benefits
How does using e-vapor products make you feel overall compared to smoking tobacco products?

How do you feel **physically** when using e-vapor products?

How does this feel when you compare it to smoking traditional cigarettes/use other tobacco?

What side effects (both good and bad) might e-vaping products have on you?
[Prompt with these specific ideas if they don't come up] nausea? Moodiness? Throat pain? Weight gain? Can breathe better? Do not cough? Have more energy?

Which side effects would be least desirable to you?

Which side effects would be most desirable to you?

Novelty/Fun factor
How much of your decision to use e-vapor products can be attributed to seeing others use them?

How much of your decision to use e-vapor products can be attributed to the social acceptability of the product?

IV. Final question *(3 minutes)*
Is there anything else we have not yet discussed that you would like to mention related to what we've been talking about?

V. Break *(5 minutes)*

Pass out surveys (face down) & pencils during break + prepare checks

Part II: Survey Administration + Discussion *(40 minutes)*

VI. Survey Testing *(18 minutes)*
We have developed a survey about e-vapor products that we would like you to take. In the first part of the survey, you will read information about e-vapor products and their features. Then, you will be asked to complete a series of comparison questions that will ask you to choose between 2 hypothetical-vapor products and select the product that you prefer the most, or decide you do not prefer either option. We will complete a practice question together to make sure you understand how to complete this section. There are XX questions and they may look the same, but please consider each one carefully. After you finish the comparison questions, you will be asked to complete questions about the survey and about yourself.

Lastly, after you finish the survey, I'm going to ask for your opinion on things like how the survey looks, if the questions are easy or hard to understand, and if we're asking the right questions. We will be using your feedback to improve this survey. Please tell us your honest opinion! We want to hear the good and the bad.

I'll let you know when you can begin the survey. When you see a stop sign at the bottom of your page, please put down your pencil and stop. We will wait until everyone reaches that point in the survey before completing the practice comparison question together.

As you are completing the survey:

Please circle any words that are unfamiliar
Please feel free to write down easier to understand words if you see anything that's too formal, not commonly used or incorrect.
Please note any errors or omissions

You May Begin the Survey

Car Example DCE

Let's walk through an example of the type of questions you will be asked to answer in the next section of this survey. These questions can be tricky to understand. Nearly everyone has bought or thought about buying a car. For this practice question we want to you to think about the various features of cars and select which car you like the most.

Let's review how to complete this type of question.
Systematically Review question with participants – ask which option they picked in why & describe trade offs

You just went through an example about how people make decisions about buying a car. Now you will be answering similar questions about e-vapor products.

Now, please continue on with the survey.

VII. Survey Debrief *(20 minutes)*

(a) What's your overall impression of the survey?
   a. Do you think any part of the survey was confusing or difficult? If so, what part or question?
   b. Which part of the survey was the easiest to complete?
   c. Did the pictures help you to understand the survey?

(b) Let's Discuss answers to Part X of the survey (see Usability questions p. 19& 20)
   a. What did you write down for questions 2 and 5?

(c) Let's look at Part 1 (beginning on p. 2)
   a. Were the instructions clear to you? How can we improve them?
   b. Look at the descriptions of e-vapor products on p. 2. What do you think about this information? What is missing? What did you like/dislike about how we presented this information?
   c. Did the car example help you complete the comparison tasks?
   d. Look at Question 3. Who selected option 1, option 2 or the "none" option? Can you explain how you decided between the different options?
   e. Here is a list of the attributes and levels that were used in the comparison questions you just completed **(pass out sheet)**
      i. What do you think about the attributes and levels we used in the questions? Are they the features you used/use to decide which e-vaping device you will use?
      ii. Do any attributes or levels need to be removed? Why?
      iii. Do any attributes or levels need to be added? Why?
   f. Throughout the survey did you have any problems with
      i. Language – Did you circle anything?
      ii. Numbers – percentages, etc.?
      iii. Layout, flipping pages, etc.
      iv. Directions – was anything confusing?
      v. Information about e-vapor products?

VIII. Overall Debrief + Provide Incentives *(2 minutes)*

Debrief Statement

Thank you so much for your help trying to understand what's important to users of electronic vapor products. We do not know much about why people are using these products but thanks to you, we now know a little more. As doctors and researchers begin to understand the health consequences of these products, both good and bad, we

believe we also need to understand the why consumers are turning to these products. You have been invaluable in this effort, and we really appreciate your time.

Distribute Checks

IX. Wrap Up

Make sure everyone exits the building

Team Debrief + documenting thoughts while ideas are fresh (30 minutes)
